# TRPV2 knockout mice demonstrate an improved cardiac performance following myocardial infarction due to attenuated activity of peri-infarct macrophages

**DOI:** 10.1371/journal.pone.0177132

**Published:** 2017-05-08

**Authors:** Michal Entin-Meer, Lena Cohen, Einat Hertzberg-Bigelman, Ran Levy, Jeremy Ben-Shoshan, Gad Keren

**Affiliations:** 1 The Laboratory of Cardiovascular Research, Department of Cardiology, Tel-Aviv Sourasky Medical Center, Tel-Aviv, Israel; 2 Department of Cardiology, Sackler Faculty of Medicine, Tel-Aviv University, Tel-Aviv, Israel; Max Delbruck Centrum fur Molekulare Medizin Berlin Buch, GERMANY

## Abstract

**Background:**

We have recently shown that the expression of the transient receptor potential vanilloid 2 channel, TRPV2, is upregulated in the peri-infarct zone 3–5 days following an acute myocardial infarction (AMI). Further analysis has demonstrated that invading monocytes maturing to macrophages merely harbor the documented elevated expression of this channel.

**Purpose:**

Assess cardiac function in TRPV2-KO mice compared to TRPV2-WT following AMI and analyze the potential involvement of TRPV2-expressing macrophages in the recovery process.

**Methods:**

TRPV2-KO or WT mice were induced with AMI by ligation of the left anterior descending artery (LAD). In another set of experiments, TRPV2-KO mice induced with AMI, were intravenously (IV) injected with WT or TRPV2-KO peritoneal macrophages in order to directly assess the potential contribution of TRPV2-expressing macrophages to cardiac healing. Cardiac parameters were obtained by echocardiography 1 day and 30 days post infarction. The relative changes in the ejection fraction (EF) and additional cardiac parameters between baseline (day 1) and day 30 were calculated and statistical significance was determined (SPSS).

**Results:**

The *in vivo* study showed that while EF was significantly decreased in the WT animals between baseline and day 30, EF was only slightly and insignificantly reduced in the KO animals. Likewise LVESD and LVESA were significantly modified exclusively in the WT animals. Moreover, intravenous administration of peritoneal WT macrophages, but not KO macrophages, significantly reduced survival of post-MI TRPV2-KO mice.

**Conclusion:**

The data suggest that knockout of the TRPV2 channel may attenuate macrophage-dependent pro-inflammatory processes and result in better cardiac recovery. TRPV2 may thus represent a novel therapeutic target for treatment of patients undergoing an acute MI.

## Introduction

Acute myocardial infarction (AMI) involves massive infiltration of inflammatory cells into the peri-infarct zone. The inflammatory response holds a key role in left ventricular (LV) remodeling and tissue repair following AMI, leading to the replacement of the necrotic myocardium with scar tissue. This process has been shown to be critically mediated by monocytes, which mature to active macrophages at the inflammatory site. Thus, monocytes have recently drawn considerable attention as a target to improve post-AMI repair [1, 2]. It has recently been demonstrated that CD14^+^- expressing macrophages predominantly accumulate at the infarct zone twelve hours- five days following the ischemic event [3]. In our previous work we have shown that 15–20% of these peri-infarct macrophages, exclusively express the noxious heat-activated Transient Receptor Potential Vanilloid 2 (TRPV2) Ca^2+^ channel on their cell surface [4]. TRPV2 is a weak Ca^2+^-selective cation channel which consists of six transmembrane regions and is described to be regulated by Insulin-Like Growth Factors [5]. Its full-length structure has recently been deciphered and it is currently recognized that it differs from TRPV1 in its overall cellular function [6, 7]. TRPV2 holds moderate to high expression in various tissues including the phagocyte and the lymphocyte subpopulations [8–10]. Interestingly, Caterina and his colleagues have previously shown that migration and phagocytosis processes are impaired in macrophages lacking this cation channel [11]. In line with this report, we have demonstrated that transfection of rat alveolar cells with siRNA to TRPV2, significantly attenuated their migratory capacity towards conditioned media of cardiomyoctes subjected to hypoxia [4]. The data suggested that the migration capacity of the activated peri-infarct macrophages towards the injured cardiomyocytes may be, at least in part, TRPV2- dependent.

In the current study we have utilized an *in vivo* model for AMI in WT versus whole body TRPV2 knockout (KO) mice in order to assess whether the lack of active TRPV2 is beneficial or detrimental in the post-MI cardiac repair processes. Moreover, in order to carefully examine the potential involvement of TRPV2-expressing macrophages in cardiac remodeling, KO mice were adoptively transferred with either WT or TRPV2-KO macrophages after MI induction and cardiac function was analyzed 30 days later. The data obtained may shed light on the potential involvement of TRPV2 in the stormy inflammatory processes taking place upon cardiac ischemia and may pave the way for the development of novel anti-inflammatory agents in order to achieve an improved recovery in patients presenting with AMI.

## Materials & methods

### Ethics statement

The study was performed following approval of The Animal Care and Use Committee of the Tel-Aviv Sourasky Medical Center (approval number: 32a-11-13) which conforms to the policies of the American Heart Association and the Guide for the Care and Use of Laboratory Animals. Surgeries were performed with isoflurane anesthesia and all efforts were made to minimize suffering. Animals were monitored 3–4 times per week for potential signs of suffering, mainly weight loss of more than 10% compared to their weight as documented on the day of initiation of the experimental procedure (day 0) and for potential significant changes in animals' behavior, mobility or body posture. Should mice have met one of these criteria; euthanasia would have been warranted on the same day in order to prevent further suffering. When the experiment was completed mice were euthanized by carbon dioxide (CO_2_) inhalation.

### *In vivo* model

TRPV2-KO or TRPV2-WT mice (a generous gift from M. Caterina, John Hopkins, USA [12]) were induced with AMI by permanent ligation of the left anterior descending artery (LAD), according to our established protocol [4, 13]. Briefly, mice were anesthetized with 2% isoflurane, intubated orally, and artificially ventilated with a respirator. A small oblique thoracotomy was performed to expose the heart. The pericardium was opened and the proximal LAD branch of the left coronary artery was ligated. Acute MI was induced in 30 mice (15/arm). Two WT animals died on the following day, probably due to a massive cardiac insult. Five other animals, four WT and one KO, were excluded from the experiment due to lack of signs for cardiac injury in the baseline echocardiography (these mice were euthanized by CO_2_ inhalation). All other animals: 9 WT and 14 KO survived until the end of the experiment (day 30) with no need for humane endpoint after being subjected to careful monitoring. Sham-operated mice (n = 5) underwent the same surgical procedure without ligation of the artery and served as controls. In order to alleviate pain, a subcutaneous (SC) injection of carprofen (5 mg/kg) was given during the surgery as well as once a day during the next three subsequent days. Cardiac parameters were obtained by echocardiography (Vevo 2100, VisualSonics, Toronto, Canada) one day and 30 days post-infarction. The relative changes in cardiac parameters between baseline (day 1) and day 30 were calculated according to the following formula:

$$\%\;of\;change=\frac{\left(value\;on\;day\;30)-(value\;on\;day\;1\right)}{\left(value\;on\;day\;30\right)}*100$$

LV end-diastolic and end-systolic volumes (LVEDV and LVESV) were automatically calculated by the Vevo cardiac software using the Simpson's formula and derived from the dimensions of the LV measured by the operator. Stroke volume (SV) was calculated as the difference between LVEDV and LVESV and cardiac output (CO) was derived from SV and heart rate. One day following the second echocardiography scan, mice were euthanized by CO_2_ inhalation and hearts were removed for further analyses.

### Isolation of peritoneal macrophages

Peritoneal fluid enriched with macrophages was aspirated from both WT and KO animals 3–4 days after injection of fluid thioglycolate (2.98 g dissolved in 100 ml of DDW; Fluka, USA) to their peritoneal cavity. The cells were then seeded on tissue culture dishes and kept in the presence of RPMI medium supplemented with 15% heat-inactivated fetal bovine serum, 5% pen/Strep and 5% glutamine and 50 μM ß-ME for up to two weeks. For the macrophage homing experiments, prior to scraping the cells with trypsin/EDTA, the cells were stained for 5 minutes at 37°C with DiI (1,1’-dioctadecyl-3,3,3’, 3’-tetramethylindocarbocyanine perchlorate) diluted 1:1000 in PBS (stock 1mg/ml in ethanol; Thermo Fisher Scientific USA) followed by five washes in PBS.

### Infusion of peritoneal macrophages

For the *in vivo* experiment involving IV injection of WT or KO macrophages, acute MI was induced in 18 mice. One mouse died on the next day, probably due to a massive cardiac insult, and two other mice were excluded from the experiment since the baseline echocardiography did not show any signs for cardiac injury. Two days following MI induction, the remaining 15 mice were IV injected with WT (n = 8) or KO macrophages (n = 7), in accordance with the peak time for macrophage accumulation at the peri-infarct zone [14]. Following anesthesia with isoflurane, mice were SC injected with carprofen (5mg/ Kg) to reduce pain. Then 150,000 WT or KO peritoneal macrophages diluted in 100 μl saline were injected into the jugular vein using a 30 G needle through a dissecting microscope, after careful removal of the underlying skin. The skin was closed by 6–0 polypropylene sutures. In the following three days, mice were SC injected with carprofen in order to minimize pain (altogether, mice were treated with carprofen for six consecutive days from the day of AMI induction). Animals' survival and well-being was monitored until day 30; at which point a second echocardiography scan was given and mice were terminated by CO_2_ inhalation.

### FACS analysis of peritoneal macrophages for CD11b expression

Cells isolated from the peritoneal cavity were stained with a PE-conjugated CD11b antibody or with its corresponding isotype control (eBioscence, USA) for 1 hour at 4°C in the dark and analyzed by flow cytometry (BD Biosciences FACSCanto II).

### Detection of local macrophages/ injected macrophages labeled with DiI

To determine whether the injected cells home into the injured myocardium, mice were intravenously (IV) injected with DiI- labeled peritoneal macrophages (150,000 cells in 100 μl saline) two days post infarction or sham-operation. The animals were terminated three days later and the LV tissues were isolated and fixed with 4% paraformaldehyde. The samples were then sliced into transverse sections and paraffinized. The blocks were sectioned at 5 μm slices. The slides were washed and mounted with DAPI Fluoromount-G (Southern Biotech) and analyzed by a fluorescent microscope (Olympus BX 51).

### Histology & immunohistochemistry of LV sections

In order to assess cardiac fibrosis, LV sections were stained with Masson’s trichrome (Sigma, St. Louis, MO) and reviewed by a light microscope. Staining with the murine monocyte/macrophage marker F4/80 was performed as previously described [4].

### Transwell migration assay

Fifty thousand peritoneal cells were seeded on top of 3 μM-pores insert (Nunc) and were allowed to migrate towards the lower part of the inserts for 4 hours at 37°C and 5% CO_2_. The inserts were then taken out and the cells that migrated to the lower side were fixed and stained with Commassie blue as previously described [4].

### Real-time analysis for brain natriuretic peptide (BNP) expression in LV lysates

RNA from the entire LV tissue of MI or sham-operated controls was extracted by phenol/chlorophorm (Biological industries, Israel). RNA extracts (500 ng) were transcribed using Verso RT-PCR Kits (ABgene, USA). A quantitative PCR was performed with the Sybr Green PCR kit (Invitrogen, Israel). The primers used for BNP were: forward 5'CCAGAGAACAGTCTTGAAGG3' and reverse 5'TCCGATCCGGTCTATCTTG3'. The relative mRNA expression of BNP was normalized to the expression of the GAPDH reference gene (forward 5’TGTGTCCFTCGTGGATCTGA3' and reverse 5'TTGCTGTTGTTGAAGTCGCAGGAG3').

### Statistical analysis

Differences in cardiac values between baseline and day 30 were analyzed by paired two-tailed Student’s t-test. Differences in cardiac parameters between WT and KO groups on day 1 or day 30 as well as the percent of change between the two groups were determined by unpaired two-tailed Student’s t-test. One-way ANOVA followed by Tukey's post-hoc test was administered to determine differences within three groups or more. Survival was determined by Kaplan Meier analysis followed by log-rank comparison (IBM SPSS statistics 22 software). Significance was set at *P*<0.05 (*p<0.05; **p<0.01; ***p<0.001). Results are expressed as means ± SE.

## Results

### Whole body TRPV2-KO mice display an improved cardiac function compared to their WT counterparts

**Fig 1:**
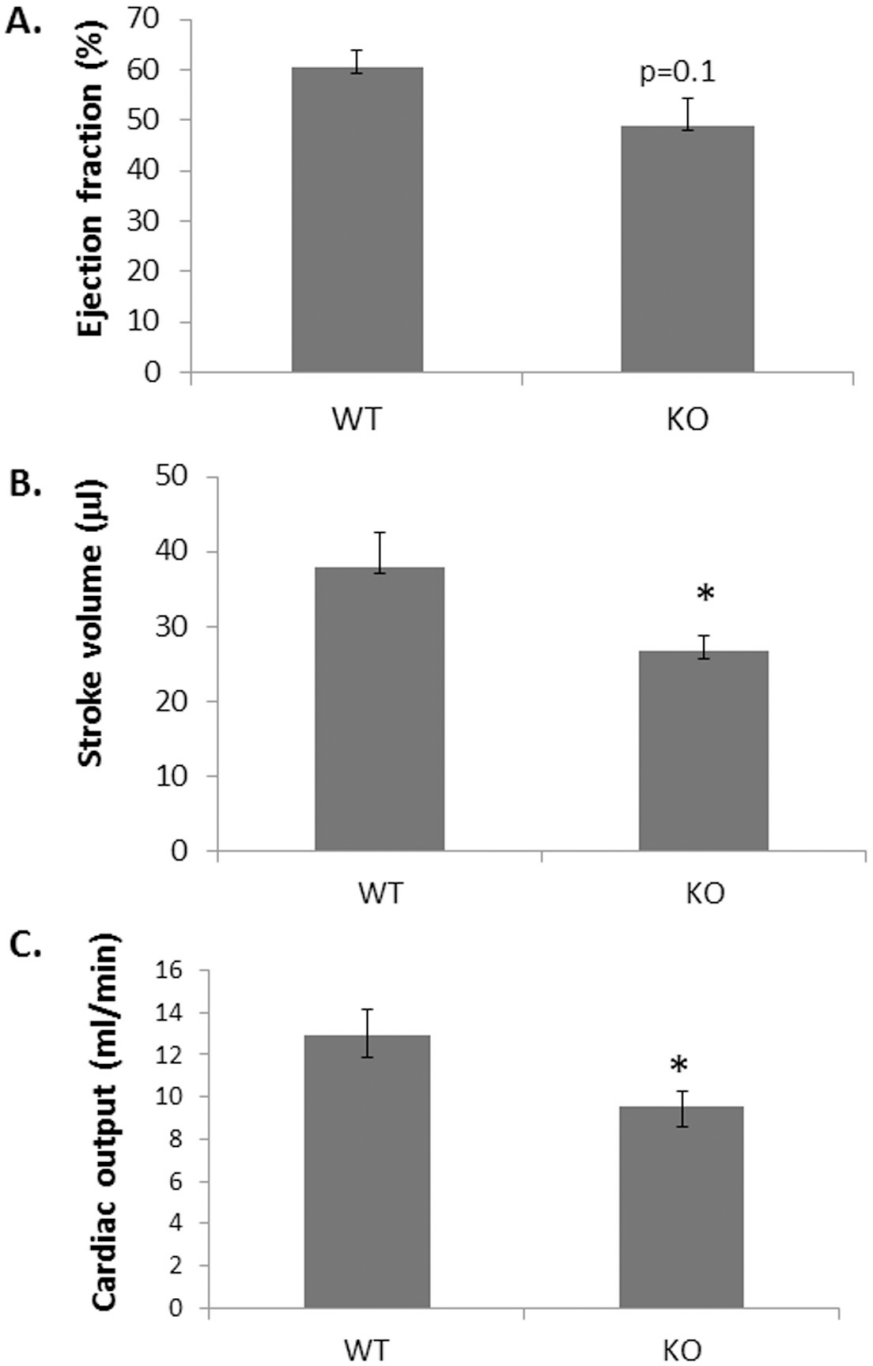
TRPV2-KO mice present a mild reduction in LV systolic function. The mean values (± SE) for EF (A), SV (B) and CO (C) of TRPV2-WT relative to TRPV2-KO mice (6 animals/ arm) according to an echocardiography scan are presented. *p<0.05.

**Fig 2:**
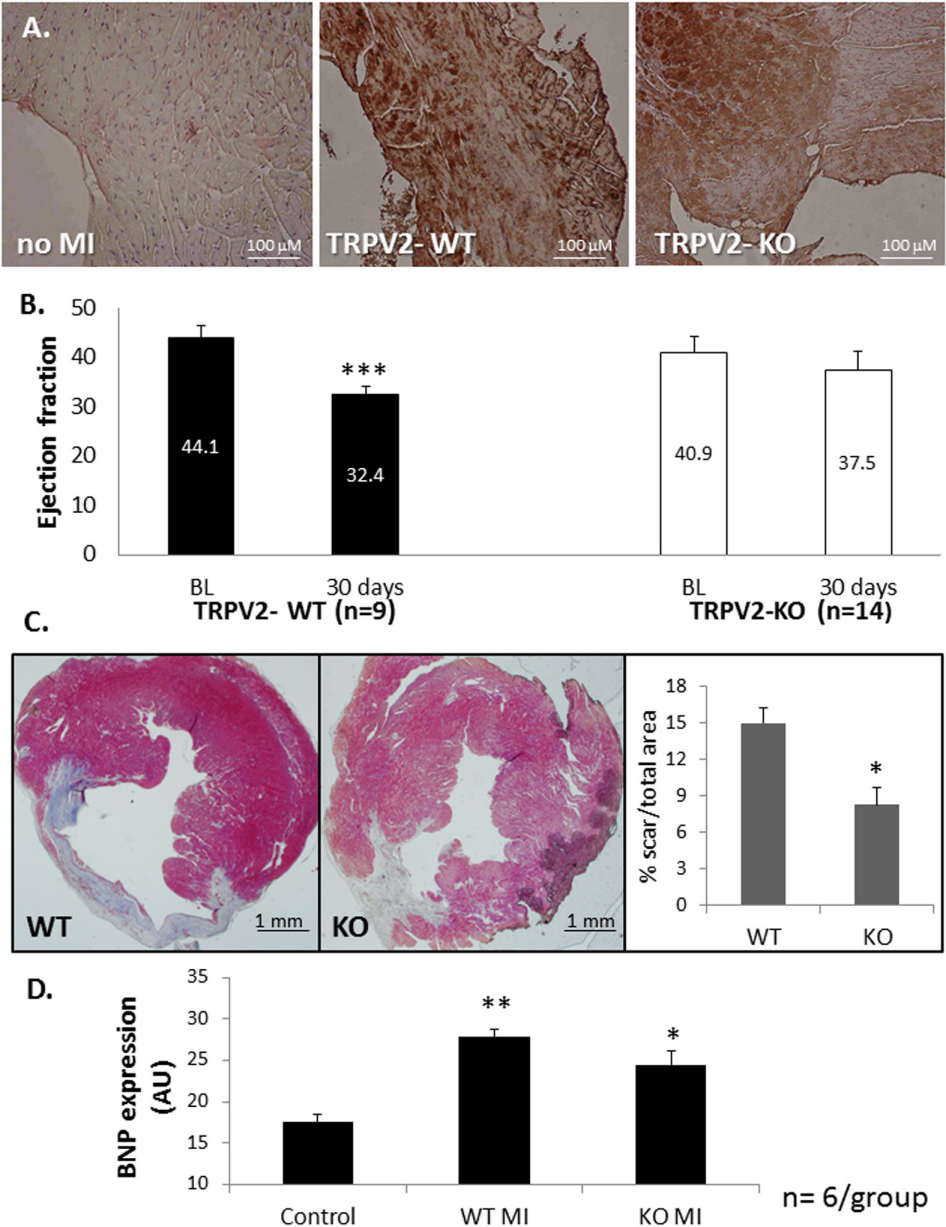
TRPV2-KO mice present an improved cardiac function following an acute MI compared to WT. TRPV2-WT and TRPV2-KO animals that underwent an acute MI 5 days earlier were tested for monocyte/macrophage expression by staining with F4/80 antibody (A). Thirty days post MI their ejection fraction relative to baseline was determined by echocardiography (B). The scar size on day 30 was analyzed by Masson's trichrome staining- representative capture and bar graph demonstrating the mean scar size of all samples (C) and BNP expression relative to sham-operated control mice was determine by real-time PCR; AU- arbitrary units (D).

First, we wished to analyze whether TRPV2-WT and TRPV2-KO animals demonstrate any differences in cardiac function as previously suggested [15, 16]. To this end, TRPV2-WT and KO male mice aged 12–16 weeks (6/arm) were studied by echocardiography. The data, given in Fig 1, indicate a mild reduction in the following values: EF (60.3 ± 3.6% in WT versus 49.0 ± 5.2% in KO mice; p = 0.1), SV (38.0 ± 4.7% relative to 26.7 ± 2.1% in WT versus KO; p = 0.05) and CO (12.9 ± 1.2 ml/min in WT versus 9.6 ± 0.7 ml/min in KO, p = 0.04). These differences may indicate that TRPV2 expression on the myocardium may be implicated in proper cardiac function. In the current study we sought to assess the potential effects of knock outing TRPV2 on the healing processes following myocardial infarction. For this purpose, TRPV2-WT and TRPV2-KO mice were induced with an acute MI followed by an echocardiography scan 1 day and 30 days post infarction. All twenty three mice in both arms, 9 WT and 14 TRPV2-KO survived between day 1, which was considered baseline (BL), and day 30 at which the experiment was terminated following the second echocardiography scan. An immunohistochemical (IHC) analysis has demonstrated monocyte/macrophage infiltration to LV sections of both WT and TRPV2-KO mice five days post infarction (Fig 2A), indicating that an impaired TRPV2 does not abolish monocyte invasion at the peri-infarct zone. Table 1 presents echocardiographic parameters of murine LV function obtained by 2-D echocardiography 1 day (BL) and 4 weeks after myocardial infarction was induced. The echocardiography analysis pointed that whole body knockout of active TRPV2 attenuated the typical LV dysfunction observed in the TRPV2-WT animals. As expected, TRPV2-WT mice exhibited worsen LV systolic function which was evident by reduced EF between baseline and day 30 (44.1 ± 2.3% versus 32.4 ± 1.8%; p<0.001). On the contrary, the EF of the TRPV2-KO mice was only slightly and insignificantly reduced (40.9 ± 3.2% versus 37.5 ± 3.7% on day 1 versus day 30, respectively; p = 0.08) (Table 1 & Fig 2B). The reduction in the EF values of the WT animals after 4 weeks was accompanied by a significant increase in the following parameters of cardiac remodeling: LV end-diastolic dimension (LVEDD), LV end-systolic dimension (LVESD), LV end-systolic area (LVESA), LV end-diastolic areas (LVEDA) and LV mass. Concomitantly, fractional area change (FAC), LV stroke volume and cardiac output were significantly reduced one month post infarction. In contrast, the TRPV2 KO animals showed only a mild increase in the following LV dilatation indexes: LVEDD, LVEDA, LVESA, with no significant change in LVESD, FAC, stroke volume or cardiac output. The degree of change between the two groups proved significant in all parameters (p<0.05), except for LV mass (p = 0.67) and a trend towards significance in endocardial FAC (p = 0.06). Altogether we found a marked effect of TRPV2 knockout on the prevention of LV dilatation and on attenuation of LV dysfunction. Histological analysis supported the echocardiographic data by demonstrating a smaller scar size in LV sections of KO animals relative to their WT counterparts (14.9 ± 1.3% versus 8.2 ± 1.4% out of the total LV cross sections in WT versus TRPV2-KO, respectively; Fig 2C). We have also assessed the mRNA expression level of the BNP gene, a known marker for an acute heart failure [17], in LV lysates of six animals per group: Sham, TRPV2-KO or TRPV2-WT (Fig 2D). As expected, both WT and TRPV2-KO mice that underwent an acute MI 30 days earlier exhibited a higher BNP expression compared to sham-operated WT controls (27.9 ± 0.9 and 24.4 ± 1.7 in WT and KO animals with acute MI compared to 17.5 ± 1.0 in sham-operated controls; p< 0.05). Interestingly, the data suggest that TRPV2-KO animals present a somewhat lower expression of BNP relative to their WT counterparts with a trend towards statistical significance (p = 0.075), supporting their improved cardiac function.

**Table 1:** Comparison of LV remodeling and function between TRPV2-WT and TRPV2-KO animals following an acute MI.

Cardiac parameter	TRPV2 WT (n = 9)	TRPV2 KO (n = 14)	p-value^2^ (unpaired t-test)
**LVEDD (mm)**			
BL	3.6 ± 0.1	3.4 ± 0.2	0.48
Day 30	4.0± 0.1	3.8 ± 0.1	0.22
% of change	15.3 ± 2.6	5.8 ± 1.7	
p-value^1^ (paired t-test)	0.01*	0.03*	0.03*^#^
**LVESD (mm)**			
BL	2.7 ± 0.1	2.7 ± 0.2	0.90
Day 30	3.2 ± 0.1	2.9 ± 0.2	0.24
% of change	27.1 ± 8.0	3.4 ± 1.5	
p-value^1^ (paired t-test)	0.02*	0.46	0.02* ^**#**^
**LVEDA (mm**^**2**^**)**			
BL	9.6 ± 0.7	11.5± 1.2	0.20
Day 30	12.8 ± 1.0	13.3 ± 0.8	0.60
% of change	29.7 ± 5.7	14.3 ± 5.0	
p-value^1^ (paired t-test)	0.008***	0.03*	0.08 ^**#**^
**LVESA (mm**^**2**^**)**			
BL	6.4 ± 1.0	6.6 ± 0.5	0.96
Day 30	9.5 ± 1.3	8.3 ± 0.6	0.35
% of change	62.5 ± 16.6	27.0 ± 8.5	
p-value^1^ (paired t-test)	0.05*	0.04*	0.04* ^**#**^
**Endocardial FAC (%)**			
BL	40.0 ± 5.1	38.8 ± 2.3	0.91
Day 30	28.6 ± 1.9	35.1 ± 3.7	0.13
% of change	-27.9 ± 5.1	-11.2 ± 6.3	
p-value^1^ (paired t-test)	< 0.0001***	0.09	0.06 ^**#**^
**Stroke volume (μl)**			
BL	26.9 ± 1.8	25.9 ± 2.7	0.30
Day 30	19.7 ± 2.1	22.3 ± 2.6	0.99
% of change	-10.0 ± 1.8	4.7 ± 4.3	
p-value^1^ (paired t-test)	0.03*	0.32	0.04*^#^
**Cardiac output (ml/min)**			
BL	11.4 ± 0.7	11.0 ± 1.1	0.34
Day 30	7.3 ± 2.2	9.9 ± 1.2	0.20
% of change	-21.1 ± 6.0	-3.3 ± 0.8	
p-value^1^ (paired t-test)	0.04*	0.56	0.05*
**Ejection fraction (%)**			
BL	44.1 ± 2.3	40.9 ± 3.2	0.49
Day 30	32.4 ± 1.8	37.5 ± 3.7	0.32
% of change	-26.2 ± 3.2	8.0 ± 6.1-	
p-value^1^ (paired t-test)	0.0001***	0.08	0.03* ^**#**^
**LV mass (mg)**			
BL	88.9 ± 7.0	91.5 ± 5.6	0.77
Day 30	105.9 ± 4.6	116.6 ± 8.4	0.34
% of change	27.0 ± 10.1	30.2 ± 9.9	
p-value^1^ (paired t-test)	0.007***	0.008***	0.67 ^**#**^

Percent (%) of change- represents the mean difference in each cardiac parameter: LVEDD, LVESD, LVEDA, LVESA, endocardial FAC, ejection fraction and LV mass (± SE) according to the following formula: [echo (day 30)- echo (day1)]/echo (day 1)*100. The % of change was calculated between BL and day 30 (two middle columns). p-value^1^ = p-value for the difference between day 1 and day 30 (paired t-test); p-value^2^ = p-value for the difference between WT and KO on day 1 and day 30 (unpaired t-test); ^#^ = p-value for the percent of change between the two groups: WT and KO (unpaired t-test). *p<0.05, **p<0.01, ***p<0.001.

### Infusion of WT, but not KO, macrophages to AMI-induced TRPV2-KO mice leads to increased mortality

**Fig 3:**
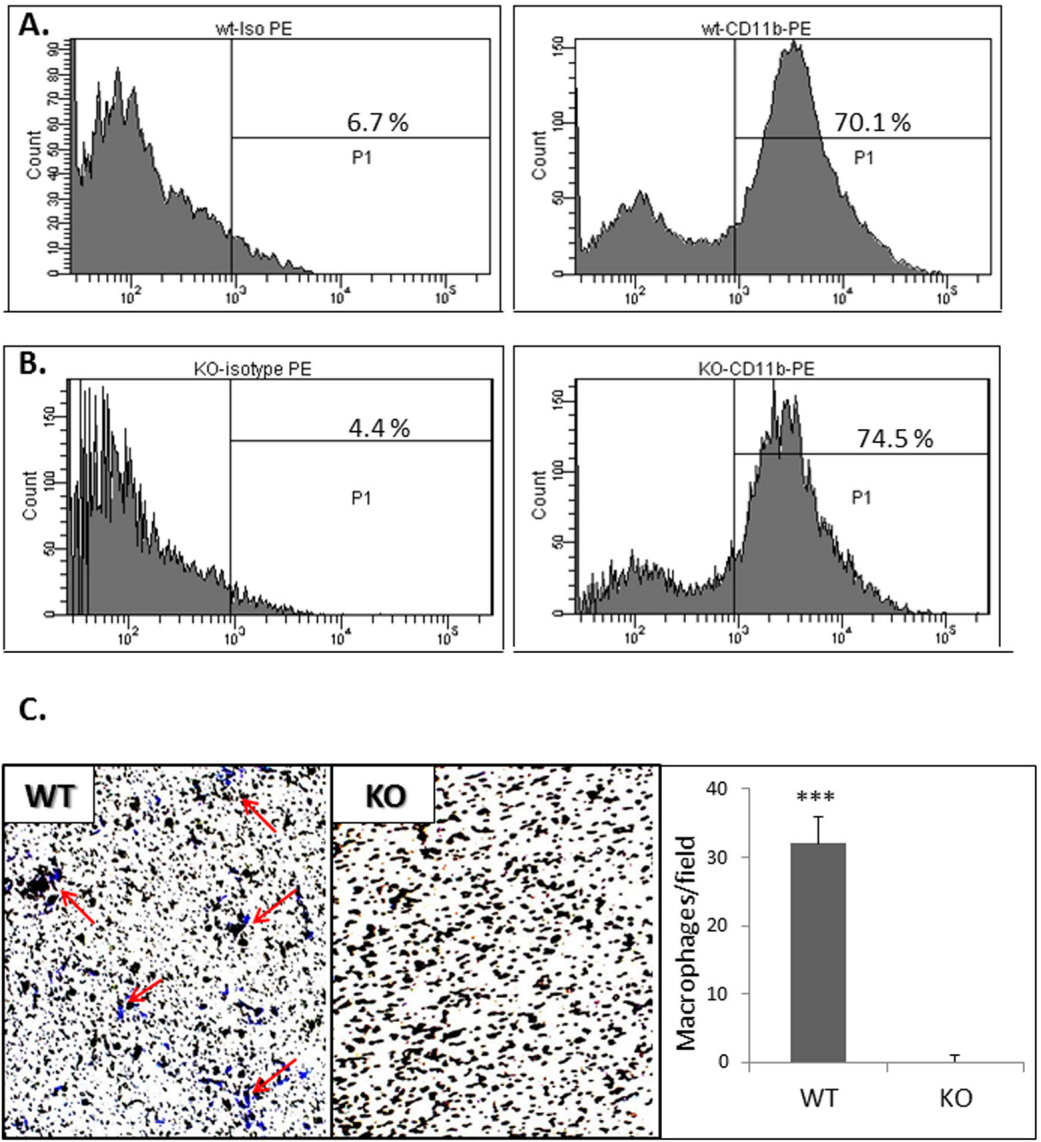
Characterization of peritoneal cells following injection of thioglycolate. Monocyte origin of the isolated peritoneal cells was confirmed by the elevated CD11b expression in both TRPV2-WT (A) and TRPV-2 KO mice (B) relative to isotype control antibody. The lack of migratory capacity of the TRPV2-KO compared to the WT-derived macrophages (representative capture & bar graph) is shown in panel C. Purple dots, some of which indicated by red arrows, represent cells that have migrated to the lower side of the inserts.

**Fig 4:**
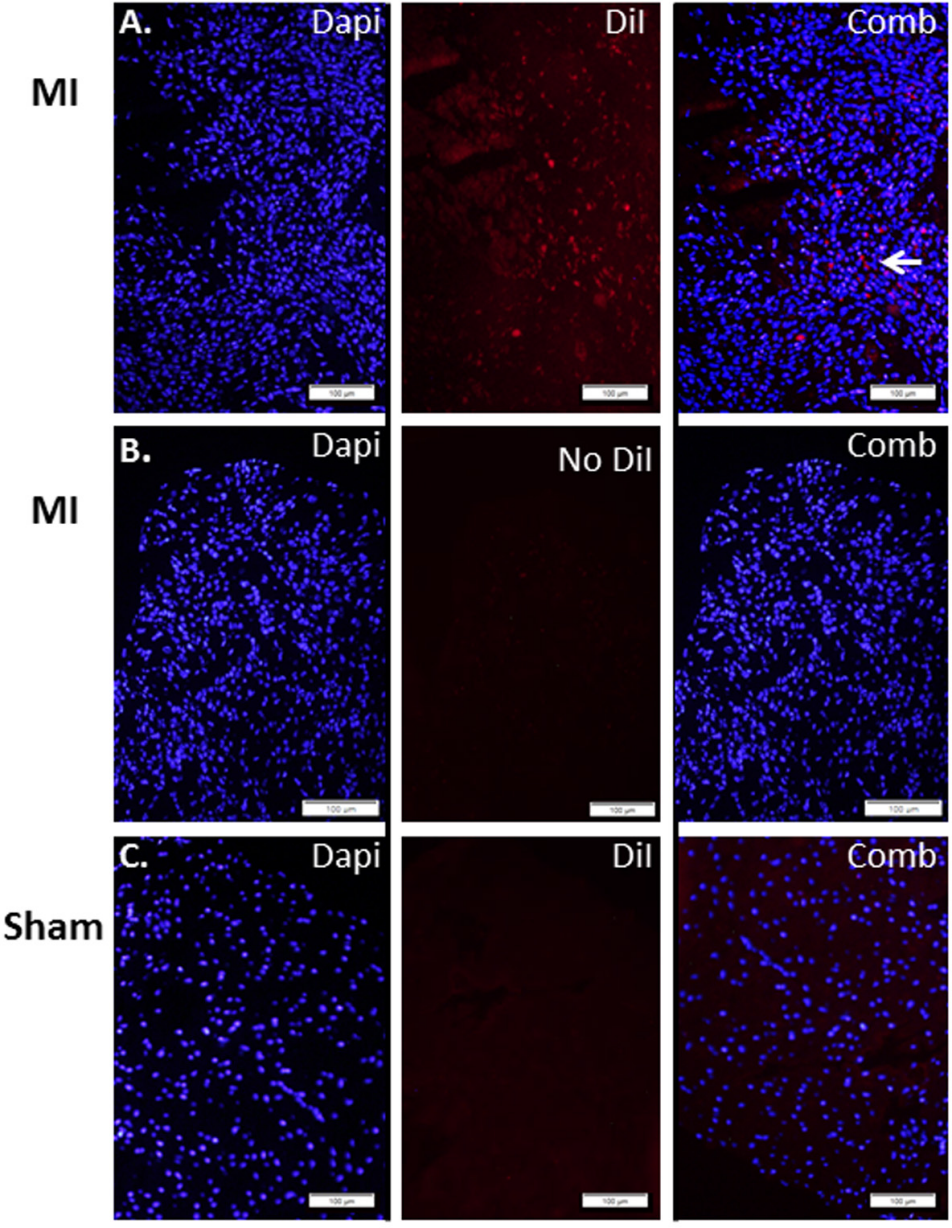
DiI- stained macrophages home at the peri-infarct zone. LV sections three days after IV injection of DiI-labeled macrophages: (A) DiI-stained macrophages injected to mice that underwent an acute MI two days earlier; (B) unstained macrophages injected to post MI animals and (C) DiI-macrophages inoculated to sham-operated control mice. Arrow points to clusters of migrating cells.

In view of these data as well as our previous report [4], we sought to investigate whether the beneficial effects on cardiac recovery following an acute MI are derived, at least in part, from the lack of active TRPV2 in the peri-infarct macrophages. To this end, we first isolated peritoneal cells from both WT and KO animals. The cells were highly enriched in CD11b expression (Fig 3A & 3B), pointing to the monocyte origin of the isolated cells. We also observed that while the TRPV2-WT macrophages presented migratory capacity towards complete DMEM medium (32 ± 4 cells/field), the TRPV2-KO macrophages were completely devoid from this capacity (0 ± 1.0 cells/field), p< 1*10^−5^ (Fig 3C). The data confirm our in vitro observations described before [4]. Altogether, the data suggest that while similar numbers of monocytes invade into the infarct zone at the acute phase post infarction (Fig 2A), mainly those monocytes that mature to TRPV2-expressing macrophages can mediate the migration capacity of the macrophages towards the injured cardiomyocytes (Fig 3C). We then wished to assess whether an intravenous administration of peritoneal macrophages two days post MI would result in their homing to the peri-infarct zone. Indeed, we observed that DiI-stained macrophages have accumulated around the infarct zone but not in remote areas, following termination three days later. No monocyte accumulation was detected in the LV sections of the sham-operated controls (Fig 4).

**Fig 5:**
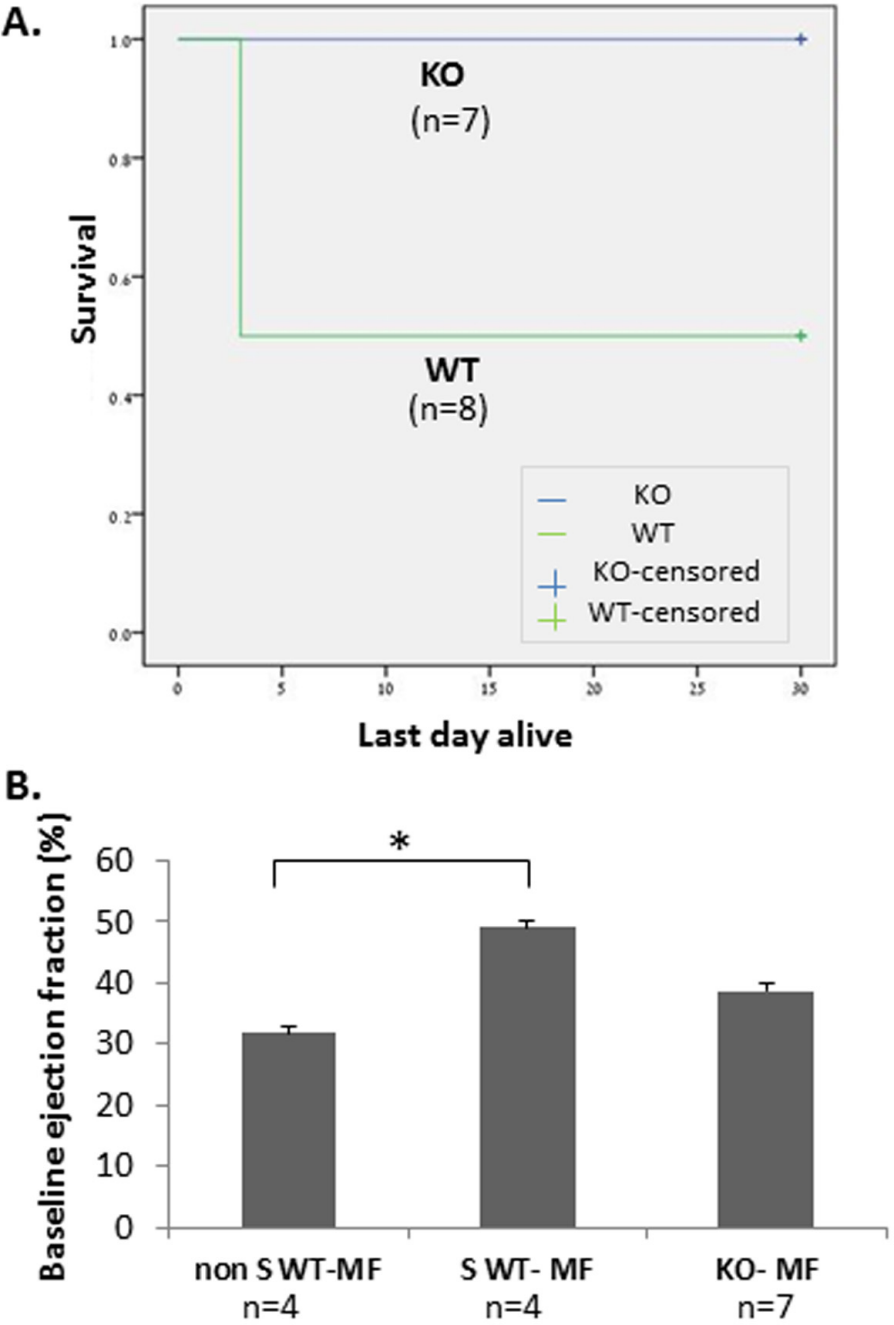
Intravenous administration of WT macrophages, but not KO macrophages, increases the mortality of post MI- TRPV2-KO animals. (A) Survival curve demonstrating the increased mortality following IV administration of WT macrophages (n = 8) compared to KO macrophages (n = 7); (p = 0.02). (B) Bar graph showing the difference in the baseline EF among surviving and non-surviving KO animals injected with WT macrophages or with KO macrophages. The differences were determined by one-way ANOVA. MF- macrophages; Non S WT MF: post MI KO animals that did not survive the administration of WT macrophages; S WT MF: mice that survived the inoculation of WT macrophages; KO MF: mice injected with KO macrophages. *p< 0.05.

**Fig 6:**
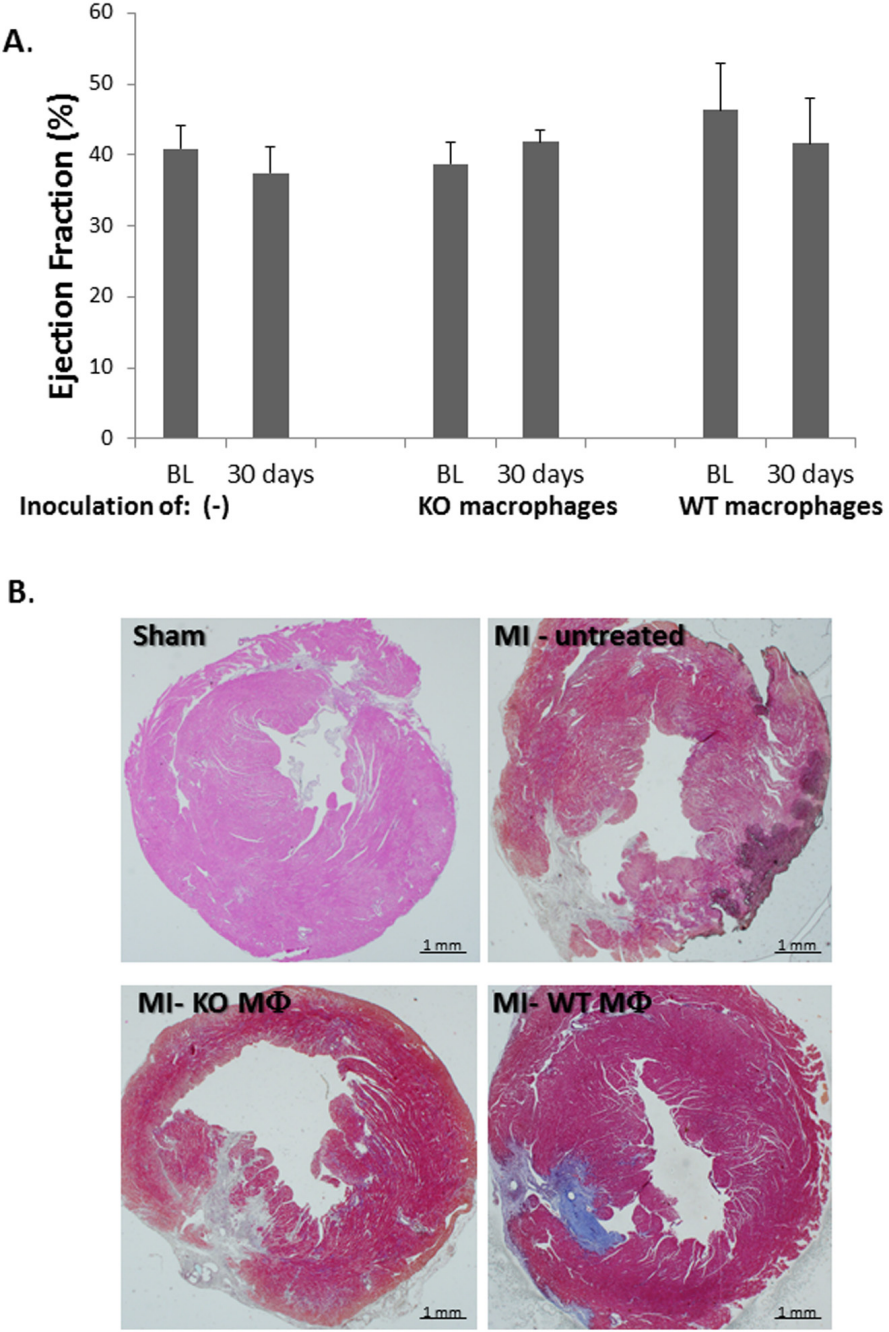
Intravenous administration of KO macrophages does not further deteriorate cardiac function of TRPV2-KO animals. (A) Ejection fraction of untreated mice (n = 14) or mice injected with KO (n = 7) versus WT macrophages (n = 4; i.e. surviving animals) in TRPV2-KO animals that underwent an acute MI one day earlier. Lack of differences between BL and day 30 for each experimental group as well as the lack of difference in the percent of change among the three groups was validated by one-way ANOVA followed by Tukey’s post-hoc correction; (B) A representative capture of the scar size of TRPV2-KO mice following one of the following procedures: sham-operation or MI induction followed by no treatment, injection of TRPV2-KO macrophages or TRPV2-WT macrophages.

Next, in order to carefully assess the potential effect of TRPV2 macrophages on cardiac recovery following an acute MI, 150,000 WT or KO macrophages were IV administered to TRPV2-KO mice, two days post infarction, via the jugular vein. Animals' survival as well as cardiac ejection fraction was documented for 30 days. Acute MI was induced in 18 mice. One mouse died on the next day, probably due to a massive cardiac insult, and two other mice were excluded from the experiment since the baseline echocardiography did not show any signs for cardiac injury. The remaining 15 mice were randomized into two experimental arms: the first one was IV injected with WT macrophages (8 mice) and the second one with KO macrophages (7 mice). Interestingly, TRPV2-KO mice inoculated with WT macrophages showed 50% mortality: four out of eight animals died up to three days following the inoculation. The remaining four mice did not show any sign of weight loss or behavioral deterioration until the end of the experiment. Conversely, all seven mice which were administered with KO macrophages survived until the end; i.e. 100% survival; p = 0.02; (Fig 5A) and did not show any sign of discomfort until termination of the experiment. Interestingly, we observed that the baseline EF values of the four mice which have survived the administration of the WT macrophages were significantly higher relative to the baseline EF of the mice that died shortly after the same inoculation (49.2 ± 4.5% versus 31.9 ± 3.8% in surviving versus non-surviving mice injected with WT macrophages, respectively; p = 0.05, n = 4/sub-group; p = 0.05). The animals injected with KO macrophages presented an intermediate initial EF of 38.8 ± 3.1% which is higher than the baseline EF of the non-surviving WT-injected mice, with a trend towards significance (p = 0.06) and somewhat lower than the baseline EF of the animals which have survived the inoculation of the WT macrophages (p = 0.2) (Fig 5B). These findings suggest that the presence of an excess number of macrophages, while some of which express an active TRPV2 channel, may be detrimental for cardiac healing. Nevertheless, the data indicate neither beneficial nor detrimental effect to the intravenous administration of KO macrophages (n = 7) compared to no injection at all (n = 14), both on systolic function (Fig 6A) and scar size (Fig 6B). Moreover, the echocardiography analysis demonstrated that the TRPV2-KO mice which have survived the administration of WT macrophages, presented similar cardiac function and scar size compared to the non-injected controls or the mice injected with KO macrophages (p>0.2; Fig 6A & 6B). It is also worthwhile to note that no major deterioration in cardiac function between day 1 and day 30 was observed in neither one of the three sub-groups of TRPV2-KO mice: untreated (n = 14), KO macrophage-treated (n = 7) or the surviving animals injected with WT macrophages (n = 4). Altogether, the data support that the excess number of macrophages *per se* may interfere with cardiac healing provided that some of the extra macrophages express functional TRPV2.

## Discussion

Myocardial ischemia profoundly changes the immune cell landscape in the heart. Within hours from onset of the ischemic event, several hundred thousand myeloid cells, mainly neutrophil and inflammatory monocytes, are recruited from the blood pool each day during the first four days after MI [18]. Accumulating data have demonstrated that if inflammatory myeloid cells are either over- or undersupplied, post MI recovery may worsen [19–21]. There is thus a great need to identify novel targets that may modulate the activity of the inflammatory macrophages that invade into the peri-infarct zone without sacrificing the steady-state haematopoiesis of the myeloid cells.

There is currently growing evidence for the potential roles of the TRPV2 channel in both innate and adaptive immune responses [22]. Numerous studies from the past few years have suggested that TRPV2 is highly abundant in macrophages upon various stimuli and that it is actually the sole member of TRPV family expressed in macrophages [8, 10, 11]. Following exposure to stimuli such as the chemotactic peptide formyl-Met-Leu-Phe (fMLP) [5] or IGF-1 [23], TRPV2 translocates from the intracellular compartments to the plasma membrane where it regulates the organization of the cytoskeletal machinery, the podosome, which is highly abundant in migrating cells. This process may be partly mediated by overproduction of TNFɑ and IL6 by the mature macrophages [10]. Moreover, PI-3 kinase signaling is known to modulate TRPV2 activity, though its effect on regulation of TRPV2 insertion into the plasma membrane is still controversial [24, 25]. Eventually TRPV2 controls the migration of the macrophages by modulating calcium entry. It has been documented that complement-mediated particle binding and phagocytosis are impaired in macrophages lacking the TRPV2 channel [11]. Moreover, in our previous study we reported that hypoxic environment results in upregulation of TRPV2 expression on the cell membrane of LV-infiltrating macrophages. We have shown that TRPV2 is significantly overexpressed in 15–20% of the peri-infarct macrophages 3–5 days post infarction, but not on circulating monocytes. The data implied that the increased expression of TRPV2 occurs locally in the ischemic zone, concomitantly with monocyte maturation to active macrophages around the infarction zone [4]. The upregulation and activation of TRPV2 on the surface of the peri-infarct macrophages may be derived from enhanced IGF-1 secretion from adjacent damaged cardiomyocytes [4, 26]. Moreover, similar to the *in vitro* data using a macrophage cell line [4], we have shown herein that TRPV2-KO macrophages are unable to migrate towards growth factors. Strikingly, we observed that TRPV2 whole body KO mice present a significantly better recovery following AMI compared to their WT counterparts and that an intravenous administration of WT macrophages but not of KO macrophages, resulted in a swift mortality. Altogether, in accordance with previous reports demonstrating the potential involvement of TRPV2 in migration and phagocytosis, the data presented herein suggest that TRPV2 can initiate a cascade for enhanced migration and phagocytosis. This cascade may be further augmented when higher numbers of total macrophages, TRPV2-expressing and non-expressing, are present at the peri-infarct zone. Thus, the findings indicate that TRPV2- mediated migratory activity of the peri-infarct macrophages, potentially due to reduced Ca^2+^ influx, may be detrimental for post MI recovery. Indeed, in line with data presented herein, it has been recently reported that TRPV2 whole-body KO mice challenged with Dextran sulfate sodium (DSS) developed a significantly reduced severity colitis compared to their WT counterparts and that the better clinical presentation of the former one was associated with a reduced number of macrophages infiltrating to the colon [27]. An additional in vitro study showed that activation of microglia, the resident macrophages of the brain and spinal cord, with the specific TRPV2 activator cannabidiol (CBD), resulted in an enhanced phagocytic activity of these cells [28]. In the case of acute MI, we anticipate that similar numbers of monocytes are recruited to the infarct area, however the migratory and potentially the phagocytic activity of the mature TRPV2-KO macrophages are moderated, leading to an improved healing.

Our data support previous reports regarding the mildly-attenuated cardiac performance in TRPV2-KO mice compared to their WT counterparts [15, 16]. Moreover, Katanosaka and his colleagues have recently reported that heart-specific elimination of TRPV2 may be critically detrimental to cardiac structure and function in mice [29]. Therefore we should take into consideration that chronic inhibition of TRPV2 may negatively affect cardiac function. Nevertheless, in order to attenuate the macrophage-mediated cardiac injury that occurs within early phase post MI, it may be prudent to use selective TRPV2 blockers at the acute phase only, i.e. up to 10 days post infarction. We anticipate that if prevention of the migratory capacity of the TRPV2- expressing peri-infarct macrophages is achieved, it may be translated into an effective treatment with probably less potential for harm to cardiac function.

### Study limitations

A) A relatively low number of animals in the adoptive transfer experiments of WT or TRPV2-KO macrophages due to roughly 50% mortality rate during the procedure itself which is performed in post-MI animals. A power analysis indicated that a total number of 20 animals, rather than15, was actually required in order to determine the magnitude of difference between the two groups with confidence (type I and type II errors ≤ 0.02; MedCalc software Version 17.2). Nevertheless, the power analysis does not take into account the variations in baseline EF among animals in each group; in particular, the differences in the WT macrophages-injected group, in which the four animals that survived the macrophage administration, had significantly higher initial EF values compared to the four ones which did not survive. Thus we believe that the number of animals we have used may suffice to draw conclusions. B) In a murine study at heart rates of 400–500 bpm, we were unable to measure reliably diastolic parameters by Doppler echocardiography, since under these conditions LV filling time is significantly shortened; on the other hand, reducing heart rate by applying deeper anesthesia may misleadingly present as reduced systolic LV function. Indeed it has been reported that since diastolic dysfunction progresses rapidly in mice, multiple different Doppler patterns may exist in the same group of mice and this may lead to misinterpretation of the stage of diastolic dysfunction [30]. C) In addition, in the current study we have demonstrated that TRPV2 whole body KO mice show better recovery following an acute MI compared to WT and pointed to the detrimental effect of TRPV2-WT macrophages on post MI healing. Nevertheless, the potential effect on inhibiting TRPV2 expression specifically in monocytes/macrophages of whole body WT animals should also be characterized. This study shall be carried out using conditional TRPV2loxP mice crossed to the macrophage LysM- Cre and will be carried out in our next in vivo studies.

## Conclusion

The data presented herein suggest that the absence of active TRPV2- expressing macrophages at the local environment of the infarcted LV results in an improved cardiac recovery after permanent LAD occlusion. The data may pave the way towards the development of novel therapeutic drugs for the multimodality management of the acute phase in post MI patients.
